# A Random Forest Model for Peptide Classification Based on Virtual Docking Data

**DOI:** 10.3390/ijms241411409

**Published:** 2023-07-13

**Authors:** Hua Feng, Fangyu Wang, Ning Li, Qian Xu, Guanming Zheng, Xuefeng Sun, Man Hu, Guangxu Xing, Gaiping Zhang

**Affiliations:** 1 Key Laboratory of Animal Immunology, Henan Academy of Agricultural Sciences, Zhengzhou 450002, China; huafeng68@outlook.com (H.F.); ln8028@163.com (N.L.); 15290032037@163.com (Q.X.); sunxuefeng2021@126.com (X.S.); human131@163.com (M.H.); xingguangxu@163.com (G.X.); 2 Public Health and Preventive Medicine Teaching and Research Center, Henan University of Chinese Medicine, Zhengzhou 450046, China; guanminzheng308@163.com; 3 Longhu Modern Immunology Laboratory, Zhengzhou 450002, China; 4 School of Advanced Agricultural Sciences, Peking University, Beijing 100871, China; 5 Jiangsu Co-Innovation Center for the Prevention and Control of Important Animal Infectious Diseases and Zoonoses, Yangzhou University, Yangzhou 225009, China

**Keywords:** peptide–protein interaction, machine learning, random forest model, affinity, prediction

## Abstract

The affinity of peptides is a crucial factor in studying peptide–protein interactions. Despite the development of various techniques to evaluate peptide–receptor affinity, the results may not always reflect the actual affinity of the peptides accurately. The current study provides a free tool to assess the actual peptide affinity based on virtual docking data. This study employed a dataset that combined actual peptide affinity information (active and inactive) and virtual peptide–receptor docking data, and different machine learning algorithms were utilized. Compared with the other algorithms, the random forest (RF) algorithm showed the best performance and was used in building three RF models using different numbers of significant features (four, three, and two). Further analysis revealed that the four-feature RF model achieved the highest Accuracy of 0.714 in classifying an independent unknown peptide dataset designed with the PEDV spike protein, and it also revealed overfitting problems in the other models. This four-feature RF model was used to evaluate peptide affinity by constructing the relationship between the actual affinity and the virtual docking scores of peptides to their receptors.

## 1. Introduction

Peptides located at the interface of protein–protein interactions (PPIs) are involved in various biological processes. Deciphering the residue composition of peptides would greatly contribute to the development of new biotherapeutics [1,2]. Since almost 15–40% of PPIs are mediated by peptides [3], research on peptides not only improves our knowledge of the functions of their receptor proteins but elucidates the biological mechanisms of downstream events in a rational way [4]. More importantly, the research could also contribute to the selection of peptides with desirable binding affinity, specificity to the interaction domain of the receptor [1,5], and the ability of modulating the PPI. Additionally, the unique characteristics of peptides, such as their small size, balanced flexibility and conformational rigidity, safety, and bio-tolerability, make them versatile candidates for various peptide-based biotherapeutics and prevention strategies [1,5], such as the PPI-interferon, recombinant epitope chimeric antigen, or nanoparticle labeled by an identified affinitive peptide, etc.

With the advancement in computer science and the improving performance of currently available computers, computational approaches have been widely employed to assist research on the peptide–protein interaction [3,6]. Currently, many well-established docking strategies are being used to analyze peptide–protein interactions [5], such as Rosetta FlexPepDock [7], CABSdock [8], HPEPDOCK [9], GalaxyPepDock [10], and rDock [11]. However, although these computational approaches have already greatly improved the efficiency of the prediction of functional peptides in recent decades, the inaccuracy of the prediction and the large consumption of computational resources are still challenging problems that need to be solved [1,3,12].Furthermore, peptide selection primarily relies on the virtual calculation of peptide–protein interaction forces. However, without considering the actual affinity of the peptides, most methods cannot accurately reflect the true affinity of the peptides to their receptors [12].

To remove these obstacles, machine learning (ML) methods have been extensively utilized to study peptide–protein interactions, benefiting peptide drug design and discovery [12]. With their high performance, and low computational resource consumption, ML-based methods could be incorporated into every step of the processes of drug discovery and development [13]. Combined with the docking process, ML has recently been trained based on both protein and ligand features or only ligand features, which was used for peptide virtual screening [14]. Additionally, ML has the ability to reduce the number of potential candidate peptides for subsequent experimental trials, thereby decreasing the risk of clinical trial failure [12,15], which could also significantly improve the time and cost efficiency of the development cycle.

**Figure 1:**
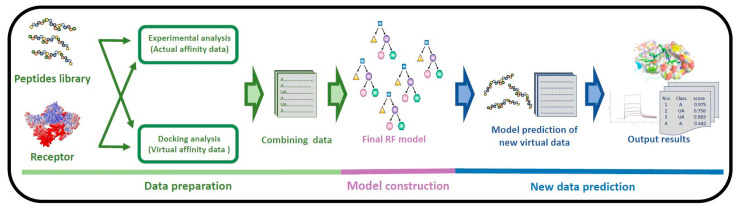
Workflow of the construction and prediction processes of the current model.

Although ML approaches have attracted increasing attention in the field of functional peptide identification, there are relatively few ML-based approaches available for identifying the peptides with binding affinity to the target protein [1]. In the current study, a series of ML models were constructed to evaluate the binding affinity types of peptides by using a dataset combining experimental actual affinity data and virtual docking score data of peptides and their expected receptors. With the best performance, the random forest (RF) model was selected for further optimization. Finally, compared with two- and three-feature RF models, a simple RF model, built using the four features with the highest important values, showed a satisfactory performance in the assessment of the test dataset, and further research on this four-feature RF model also showed its potential in predicting an unknown PEDV peptide dataset. In summary, the current study provides a promising RF model for evaluating the actual affinity of peptides to their receptors, which could benefit the future selection of affinitive peptide and peptide drugs. A schematic diagram of the construction and prediction of the optimal RF model is shown in Figure 1. The model is available at http://www.peptide-ligand.cn/index.php/virtual-screening-of-peptides/ (accessed on 1 April 2023).

## 2. Results

### 2.1. Dataset Characterization

In the current study, all 194 peptides were tested ten times by virtual prediction (rDock) or experimental methods (surface plasmon resonance, SPR). Then, an initial dataset with 1940 cases from all ten-time analyses was constructed, which included all the information of the features referring to peptides and their expected receptors. After removing some basic information about the peptides and the features with too many zero values, all the data from the 13 different features were scaled and centered to facilitate the learning process of the algorithms employed in the present study.

### 2.2. Algorithm Selection and Feature Importance

**Figure 2:**
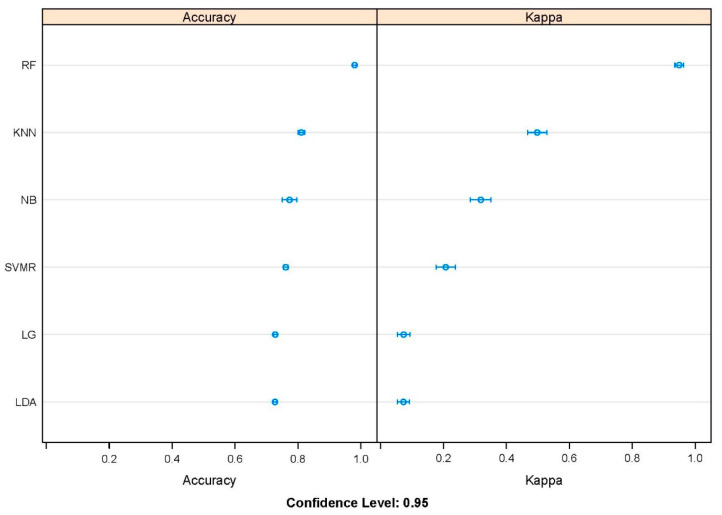
Performance comparison of different machine learning algorithms based on Accuracy and Kappa values.

To quickly get an idea of which algorithm was more suitable for the obtained data, the Accuracy and Kappa values of the different ML algorithms employed in the current study were compared. As shown in Figure 2, the Accuracy (0.98) and Kappa (0.95) values of the random forest classifier were superior to those of the other algorithms (Accuracy < 0.81 and Kappa < 0.50) at the 95% confidence level.

**Figure 3:**
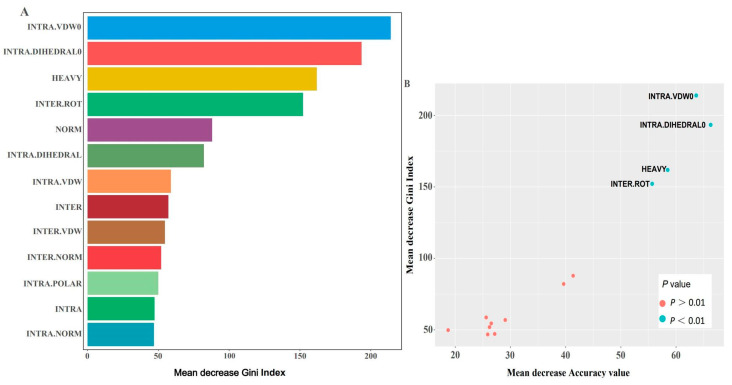
Relative importance of all features in current study. (**A**) Feature importance based on mean decrease in Gini index. (**B**) Relationship between mean decrease in Accuracy and mean decrease in Gini index grouped by *p* value.

Since the evaluation of feature scores is time-consuming for the docking process, the more important features were selected by evaluating the importance of all obtained features in alleviating the computational burden. As shown in Figure 3A, the importance of each feature was ordered by the mean decrease in Gini index, and only the first four features, including INTRA.VDW0, INTRA.DIHEDRAL0, HEAY, and INTER.ROT, had an importance higher than 100, implying that these features may play a key role in the performance of the model. Moreover, further exploration of the relationship between mean decrease in Gini index and mean decrease in Accuracy value (Figure 3B) showed that the first four important features with *p* values lower than 0.05 were clearly separated from the other features, which further confirmed the critical roles of these features in model classification. Then, these features were selected for a further modeling process, while the remaining features were removed from the dataset.

### 2.3. Construction of RF Model

Afterward, three RF models were built by using different numbers (four, three, and two) of the important features. For each model, the best mtry was optimized, and the values of Accuracy, Kappa, Sensitivity, Specificity, and AUC under the best mtry are shown in Table 1. Almost no difference was observed among the RF models built using different numbers of important features upon evaluation of the TrS. Furthermore, the performance of the four-feature model was closer to that of the two-feature model.

**Figure 4:**
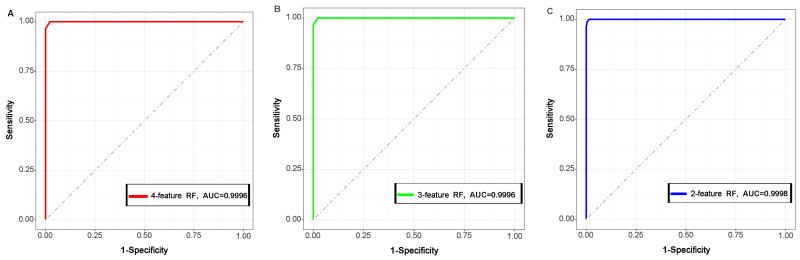
The ROC plot for 4-feature (red (**A**)), 3-feature (green (**B**)), and 2-feature (blue (**C**)) models.

Then, all the RF models were further tested by dataset InT, and the Accuracy, Kappa, Sensitivity, Specificity, F1 score, and MCC value of these models were calculated. All values of these model evaluators listed in Table 2 were higher than 0.95 for each of the models, which implied that a high performance of these RF models was established in the current study. Although the performance was not significantly different between these RF models, the model built using INTRA.VDW0 and INTRA.DIHEDRAL0 with an mtry value of 2 showed a slightly better performance than the others. And interestingly, the four- and three-feature models showed the same performance on InT. The ROC curves for each model are shown in Figure 4, and a higher AUC value for the two-feature model was also observed compared with the others.

### 2.4. Performance of RF Model on Independent Data

To further confirm the performance of the obtained RF models, the necessary features employed by the RF model of a series of peptides, designed based on the PEDV spike protein, were evaluated by rDock as described in Section 4, and were further used to simulate an independent dataset totally unknown to the established models. The actual affinity type (A and UA) was also classified based on the SPR results, which were used to verify the prediction of the RF models. As shown in Table 3, the accuracies of these RF models were 0.714, 0.661, and 0.607, respectively, for the four-, three-, and two-feature models. With the incorporation of more features, the constructed models showed an increasing trend in TP rate (from 0.668 to 0.809) but a declining trend in TF rate (from 0.333 to 0.222). Furthermore, all the constructed models performed better on active class prediction than on inactive class prediction. Finally, the RF model built with all four important features was selected as the optimal model.

## 3. Discussion

With the advancement of computational methodologies, machine learning algorithms have become attractive methods and are widely used in the research of protein–peptide interactions, which could benefit the development of new peptide-based therapeutics and significantly reduce the time and cost of this process [6,16]. Currently, although some peptide docking methods for evaluating protein–peptide interactions are well established, these methods mainly depend on a docking method and linear scoring system to predict the interaction between the peptides and their expected receptors, leading to inconsistencies between the predicted and actual affinity properties of the peptides to their expected receptors. Therefore, to solve this problem and construct the relationship between actual affinity and virtual predicted data, a random forest model for evaluating the actual affinity of peptides to their expected receptors was constructed for the first time in the current study by using datasets combining the actual affinity information with the virtual docking data of peptides and their receptors. In addition, this model can be used for affinitive peptide screening based on the data of peptide–protein docking features.

In the current study, all features representing the interaction between peptides and their receptor proteins, IgG and Aβ-42, were scored ten times using the rDock program. Since the scores of some features were obviously different among the ten-replicate analysis for each peptide, all the predicted results of these analyses were kept to avoid missing any unpredictable factors during the following model construction. In addition, to construct the connection between the virtual feature scores and the actual peptide affinity during the process of model construction, all the peptides were further synthesized and tested by SPR to obtain their actual KD value to their receptors. Since all data were generated from a standalone platform, the current study did not employ data from other online sources to ensure the integrity, reliability, and trustworthiness of the data.

As a popular ML algorithm, the random forest algorithm has been widely used in research on the peptide–protein and/or protein–protein interaction and has shown superior ability (Accuracy) compared with the other algorithms in distinguishing different cases [2,3,17,18,19,20,21,22]. In the current study, a rough comparison of the different algorithms mentioned in Materials and Methods was performed, and the RF algorithm with the highest performance (Accuracy = 0.98 and Kappa = 0.95) was selected as the most powerful method for the following optimization.

The feature importance (mean decrease in Gini index and mean decrease in Accuracy value) not only represented the contribution of each feature to the performance of the model but also indicated which features played a crucial role in defining the affinity of the peptide in the present study. Of the 13 features, 4, including INTRA.VDW0, INTRA.DIHEDRAL0, HEAVY, and INTER.ROT, were more important than the others, and their *p* values (*p* < 0.01) indicated that they were more often used in the current RF model in a random way [23]. Interestingly, it seems that the scores for each peptide obtained from the ten-replicate analyses of the four important features were almost the same and more stable compared with the scores of the other features, which may explain why these four were more important than the others to some extent. These results also implied that the affinity of the peptides to their receptors was mostly dependent on these four features of peptides. Since evaluating all the features (*n* = 16) of the interaction between the peptides and their expected receptors using the rDock software is a time-consuming task, the selection of the 4 most important features could improve the docking efficiency, simplify the following model construction, guarantee Accuracy, and save time on subsequent model prediction, while lowering the risk of the possible curse of dimensionality [18,24].

We then compared the different RF models constructed using different numbers of the four important features. As shown in Table 1, there was almost no difference among the models in Accuracy, Kappa, Sensitivity, Specificity, and AUC, although the performance of the four-feature RF model was slightly better than the others. The obtained RF models also showed a high performance in classifying the cases of InT, with all evaluator values for each model higher than 0.95. Strangely, the Accuracy, Kappa, Sensitivity, Specificity F1 score, and MCC value were lower in the four-feature model than the two-feature model. In addition, the ROC curve and AUC value, significant metrics of the machine learning model [18,25], also indicated a slightly better performance of the two-feature model than the others, which may indicate an over- or under-fitting problem.

By using a totally new dataset generated based on the PEDV spike protein, a further performance evaluation of the models was carried out to simulate the unknown data prediction. The results indicated that the four-feature model with the highest Accuracy of 0.714 performed better than the three- and two-feature models with an Accuracy of 0.661 and 0.607, respectively; these results also implied an overfitting problem in the low-feature models when testing InT. Further analysis showed that the performance of all obtained models was relatively better on class A prediction than class UA prediction, which may be explained by the imbalanced distribution of the two classes in the dataset used in model construction, as described in previous studies [26]. Although achieving an accurate prediction of active peptide is important for a peptide affinity prediction model, and the optimal four-feature model in the current study also showed a high TP rate at 0.809, more inactive cases still need to be included in future model construction to further improve the Accuracy of the current RF model.

## 4. Materials and Methods

### 4.1. Dataset Collection

Our research group screened a series of peptides against immunoglobulin G (IgG, *n* = 115) and amyloid β-protein 42 (Aβ-42, *n* = 79) [27,28,29]. All peptide sequences, their receptor proteins, and affinity information of all peptides to their receptors are listed in Supplementary Table S1 and were retrieved from the Peptide Ligand Database (DPL2: http://www.peptide-ligand.cn, accessed on 1 November 2022) [30]. All peptides (ID for Aβ-42: DPL_1069-DPL_1147; ID for IgG: DPL_1149-DPL_1263) were synthesized by Gill (Shanghai, China) with >95% purity and tested by HPLC.

#### 4.1.1. Affinity Assay between Peptides and Proteins by SPR

Equilibrium dissociation constants (KD) between all 194 (115 + 79) peptides and their receptor proteins were determined by SPR using the Biacore X100 instrument [31]. Proteins were coupled to the CM5 chip by the EDC/NHS(1-Ethyl-3-(3′-dimethylaminopropyl) carbodiimide/*n*-Hydroxysuccinimide) method (GE HealthCare, Chicago, IL, USA), followed by running HBS-EP (HEPES Buffered Saline–EDTA–Surfactant P20) buffer (GE HealthCare, USA) that flowed through the entire pipeline. The peptide was diluted in six different concentrations with HBS-EP buffer and flowed into the machine from low to high, and its resonance signal change was detected separately. In each cycle, the peptide solution flowed through the chip at a constant flow rate of 30 μL/min for 120 s, and the peptide reacted fully with the protein, followed by HBS-EP buffer flowing through the chip at the same rate for 120 s, and the peptide was partially dissociated from the protein. Finally, the peptides bound to the proteins were completely eluted off with 0.25% SDS solution for the next cycle until all peptides were detected. The KD between each peptide and protein was calculated and analyzed using Biacore X100 Evaluation Software (Version 2.1) to determine its affinity.

#### 4.1.2. Structure Preparation

Based on the sequences of the peptides mentioned above, all peptide structures were generated by Cyclops [32] with SDFile format (SDF). The crystal structures of IgG (PDB ID:5U4Y) and Aβ-42 (PDB ID:6SHS) proteins were downloaded from PDB (Protein Data Bank, https://www.rcsb.org/ (accessed on 20 December 2022)). The structure file was subjected to side chain repair, hydrogenation, and charge addition using iBabel (v4.0) [33] to ensure the integrity of the protein. The final mol2 file was generated for subsequent molecular docking.

#### 4.1.3. Molecular Docking

The interactions between all 194 selected peptides with IgG or Aβ-42 were further analyzed 10 times each using rDock software (v2013.1) [11]. All peptide–protein interaction complex structures and related information were searchable in DPL2. The entire molecular docking process was carried out in strict accordance with rDock’s operational guidelines. The first step defined the system in terms of reference peptide ligands, followed by the generation of a cavity, and finally molecular docking was performed as required.

A total of 16 features of the interactions, representing the scores of peptide–receptor interactions, relative energy of the ligand conformation, relative energy of the flexible regions of the active site, and non-physical restraint functions, were evaluated and scored. After removing the features that scored 0 for most peptides, 13 features were left (as shown in Supplementary Table S1).

Combining the results of SPR and docking, a threshold, indicating the lowest affinity between the peptides and their receptors was set at around KD = 1 × 10^−5^, based on which all the peptides were classified into two classes: active (A, KD ≤ 1 × 10^−5^) and inactive (UA, KD > 1 × 10^−5^). Then, a dataset (Supplementary Table S1) was constructed by combining the peptide ID, sequence information, receptor information, KD value, class information, and all the scores of the 13 features for each peptide from all 10 dock analyses, which contained 1400 A and 540 UA.

### 4.2. Pre-Selection of Different ML Algorithms

By using all obtained data, a series of popular supervised machine learning algorithms for classification, including Logistic Regression (LG), Linear Discriminate Analysis (LDA), k-Nearest Neighbors (KNN), Naive Bayes (NB), Support Vector Machines with radial kernel (SVMR), and random forest (RF), were preliminarily trained using R package caret (v6.0) [34] in R under the default parameters of each algorithm. In addition, a 10-fold cross-validation (CV) of the training dataset was employed in the training processes to estimate model performance, which was repeatedly performed 10 times. The Accuracy and Kappa values generated from the training process were extracted and employed to compare the performance of the different algorithms. Kappa values range from 0 to 1 and indicate the degree of consistency between the model predictions and true values. As shown in Figure 2, the RF model was superior to its competitors and showed better potential for handling the current data.

### 4.3. Selection of Important Features

The rank of the importance of each feature was correlated with the Accuracy of the model performance and indicated how the features affected the affinity between the predicted peptides and their expected receptors. The importance of all the obtained data was analyzed using an RF algorithm from R package randomForest (v4.7) [35], with 500 trees; and to avoid overfitting problems, 4 ($\sqrt{n}$ ≈ 3.6) features were selected for parameter mtry, which represents the number of variables selected and tested at each split randomly. Then, mean decrease in Gini index and mean decrease in Accuracy value, which indicate the importance of the tested features, were calculated. The *p* values of each feature were also evaluated using R package rfPermute (v2.5) package [36], which indicates the frequency of the features used by the algorithm.

### 4.4. RF Model Reconstruction Using the Important Features

The features with a significant influence (*p* < 0.05) on important values from the dataset were then extracted and further split into a training dataset (TrS, with 70% data) and an independent test dataset (InT, with 30% data). In detail, there were 1358 peptide data in TrS, including 993 for A and 365 for UA, whereas the remaining data (582) were left for InT, including 407 for A and 175 for UA.

Then, the RF algorithm was used to learn the data patterns in TrS using the caret package, and different RF models were constructed with a gradually decreasing number of important features by continuous exclusion of features with low importance. To avoid overfitting issues, 10-fold cross-validation was implemented 10 times, and mtry was also optimized for each model based on the selected features during the training process. For each model optimization process using different important features, the optimal models were selected and compared with different evaluators, including Accuracy, Kappa, Sensitivity, Specificity, and the values of area under the curve (AUC) of Receiver Operating Characteristic (ROC), which were generated from the training process.

### 4.5. Performance Evaluation of the Constructed Model

After the selection of the final model, InT was used to test the performance of the model. The Sensitivity, Specificity, Accuracy, F1 score, and Matthews’s correlation coefficient (MCC) of these models were evaluated by the following equations using the ratios of true positive (TP), false positive (FP), true negative (TN), and false negative (FN) of the selected models:

$$Sensitivity=\frac{TP}{TP+FN}\times 100\%\;Specificity=\frac{TN}{TN+FP}\times 100\%$$

$$Accuracy=\frac{TP+TN}{TP+TN+FP+FN}\times 100\%\;F1=\frac{2TP}{2TP+FP+FN}$$

$$MCC=\frac{TP\times TN-FP\times FN}{\surd (TP+FP)\times (TP+FN)\times (TN+FP)\times (TN+FN)}$$

Moreover, ROC curves of the selected RF models were plotted based on the sensitivity and specificity using R package pROC (v1.18) [37], and the AUC for each class was also calculated to evaluate the model performance.

### 4.6. Performance of the RF Models on an Unknown Peptide Dataset

Following the methods described above, a totally new dataset was constructed to further verify the performance of the constructed model. Briefly, a total of 56 six-amino-acid peptides (ID: DPL_1013-DPL_1068) were designed based on the porcine epidemic diarrhea virus spike (PEDV S) protein and were evaluated by rDock using the PEDV S crystal structure file (PDB ID:6U7K) to obtain the scores of the important features needed for the model prediction. Meanwhile, the PEDV peptides were synthesized and tested by SPR successively, and the obtained KD values were further used to define the class of each peptide based on the criteria described above. Finally, the new 6-amino-acid peptide dataset for PEDV S combining all scores of the important features and class information was built to further test the predictive correctness of the RF model (Supplementary Table S2).

All figures referred to in the current study were plotted using R package ggplot2 (v3.4) [38].

## 5. Conclusions

The peptide–protein interaction has been considered key to explaining many biological processes, and further exploring the mechanism of this interaction could promote the development of biotherapeutics. In the current study, a random forest model for predicting the affinity between peptides and their expected receptors was built by using four important features. To the best of the authors’ knowledge, this is the first model constructed by using data combining experimental affinity with virtual docking. Compared with two- and three-feature models, a better performance of the optimal four-feature model was observed when testing on InT, which was further confirmed when evaluated on an unknown PEDV peptide dataset. However, the relatively small number of inactive cases used in the current study lowered the model performance on inactive peptide classification, so more effort is needed to optimize the current model for better classification performance. In conclusion, the current study constructed a prospective RF model using actual affinity and virtual docking data to classify peptides with or without affinity, which may significantly improve the selection efficiency of affinitive peptides and contribute to the research on small peptide drugs.

## Figures and Tables

**Table 1:** Performance comparison of RF models built using different numbers of the selected important features.

Selected Variables	mtry	Accuracy	Kappa	Sensitivity	Specificity	AUC
INTRA.VDW0 INTRA.DIHEDRAL0 HEAVY INTER.ROT	4	0.9915	0.9778	0.9932	0.9858	0.9997
INTRA.VDW0 INTRA.DIHEDRAL0 HEAVY	3	0.9902	0.9753	0.9934	0.9817	0.9995
INTRA.VDW0 INTRA.DIHEDRAL0	2	0.9912	0.9781	0.9978	0.9743	0.9997

**Table 2:** Performance comparison of RF models built using different numbers of important features.

Selected Variables	Accuracy	Kappa	Sensitivity	Specificity	F1	MCC
INTRA.VDW0 INTRA.DIHEDRAL0 HEAVY INTER.ROT	0.9880	0.9707	0.9928	0.9762	0.9916	0.9707
INTRA.VDW0 INTRA.DIHEDRAL0 HEAVY	0.9880	0.9707	0.9928	0.9762	0.9916	0.9707
INTRA.VDW0 INTRA.DIHEDRAL0	0.9897	0.9737	1	0.9623	0.9930	0.9741

**Table 3:** The performance of the RF models built by using different numbers of important features on an unknown PEDV dataset.

Models	Class		Prediction Affinity (*n*)		Accuracy
			A	UA	
4-feature model	Actual affinity (*n*)	A	760	180	0.714
		UA	140	40	
3-feature model		A	680	260	0.661
		UA	120	60	
2-feature model		A	620	320	0.607
		UA	120	60	

## Data Availability

Extra information about the data employed in this article is available in DPL2 (http://www.peptide-ligand.cn, accessed on 1 November 2022).

## Supplementary Materials

The following supporting information can be downloaded at https://www.mdpi.com/article/10.3390/ijms241411409/s1.

## References

[1] Lei Y., Li S., Liu Z., Wan F., Tian T., Li S., Zhao D., Zeng J. (2021). A deep-learning framework for multi-level peptide–protein interaction prediction. Nat. Commun..

[2] Johansson-Åkhe I., Mirabello C., Wallner B. (2019). Predicting protein-peptide interaction sites using distant protein complexes as structural templates. Sci. Rep..

[3] Johansson-Åkhe I., Mirabello C., Wallner B. (2020). InterPep2: Global peptide–protein docking using interaction surface templates. Bioinformatics.

[4] Caporale A., Adorinni S., Lamba D., Saviano M. (2021). Peptide-Protein Interactions: From Drug Design to Supramolecular Biomaterials. Molecules.

[5] Lee A.C., Harris J.L., Khanna K.K., Hong J.H. (2019). A Comprehensive Review on Current Advances in Peptide Drug Development and Design. Int. J. Mol. Sci..

[6] Tripathi N.M., Bandyopadhyay A. (2022). High throughput virtual screening (HTVS) of peptide library: Technological advancement in ligand discovery. Eur. J. Med. Chem..

[7] London N., Raveh B., Cohen E., Fathi G., Schueler-Furman O. (2011). Rosetta FlexPepDock web server—High resolution modeling of peptide-protein interactions. Nucleic Acids Res..

[8] Bielza C., Larrañaga P. (2014). Discrete Bayesian Network Classifiers: A Survey. ACM Comput. Surv..

[9] Zhou P., Jin B., Li H., Huang S.-Y. (2018). HPEPDOCK: A web server for blind peptide–protein docking based on a hierarchical algorithm. Nucleic Acids Res..

[10] Lee H., Heo L., Lee M.S., Seok C. (2015). GalaxyPepDock: A protein–peptide docking tool based on interaction similarity and energy optimization. Nucleic Acids Res..

[11] Carmona S.R., Alvarez-Garcia D., Foloppe N., Garmendia-Doval A.B., Juhos S., Schmidtke P., Barril X., Hubbard R.E., Morley S.D. (2014). rDock: A Fast, Versatile and Open Source Program for Docking Ligands to Proteins and Nucleic Acids. PLoS Comput. Biol..

[12] Patel L., Shukla T., Huang X., Ussery D.W., Wang S. (2020). Machine Learning Methods in Drug Discovery. Molecules.

[13] Gupta R., Srivastava D., Sahu M., Tiwari S., Ambasta R.K., Kumar P. (2021). Artificial intelligence to deep learning: Machine intelligence approach for drug discovery. Mol. Divers..

[14] Gupta P., Mohanty D. (2021). SMMPPI: A machine learning-based approach for prediction of modulators of protein-protein interactions and its application for identification of novel inhibitors for RBD:hACE2 interactions in SARS-CoV-2. Brief. Bioinform..

[15] Bukhari SN H., Jain A., Haq E., Mehbodniya A., Webber J. (2022). Machine Learning Techniques for the Prediction of B-Cell and T-Cell Epitopes as Potential Vaccine Targets with a Specific Focus on SARS-CoV-2 Pathogen: A Review. Pathogens.

[16] Kumari M., Subbarao N. (2021). Deep learning model for virtual screening of novel 3C-like protease enzyme inhibitors against SARS coronavirus diseases. Comput. Biol. Med..

[17] Jabeen A., de March C.A., Matsunami H., Ranganathan S. (2021). Machine Learning Assisted Approach for Finding Novel High Activity Agonists of Human Ectopic Olfactory Receptors. Int. J. Mol. Sci..

[18] Danishuddin, Kumar V., Parate S., Bahuguna A., Lee G., Kim M.O., Lee K.W. (2021). Development of Machine Learning Models for Accurately Predicting and Ranking the Activity of Lead Molecules to Inhibit PRC2 Dependent Cancer. Pharmaceuticals.

[19] Jana T., Ghosh A., Das Mandal S., Banerjee R., Saha S. (2017). PPIMpred: A web server for high-throughput screening of small molecules targeting protein–protein interaction. R. Soc. Open Sci..

[20] Abella J.R., Antunes D.A., Clementi C., Kavraki L.E. (2020). Large-Scale Structure-Based Prediction of Stable Peptide Binding to Class I HLAs Using Random Forests. Front. Immunol..

[21] Wang C., Zhang Y. (2017). Improving scoring-docking-screening powers of protein-ligand scoring functions using random forest. J. Comput. Chem..

[22] Liu S., Alnammi M., Ericksen S.S., Voter A.F., Ananiev G.E., Keck J.L., Hoffmann F.M., Wildman S.A., Gitter A. (2019). Practical Model Selection for Prospective Virtual Screening. J. Chem. Inf. Model..

[23] Machado G., Vilalta C., Recamonde-Mendoza M., Corzo C., Torremorell M., Perez A., VanderWaal K. (2019). Identifying outbreaks of Porcine Epidemic Diarrhea virus through animal movements and spatial neighborhoods. Sci. Rep..

[24] Wei Y., Li J., Qing J., Huang M., Wu M., Gao F., Li D., Hong Z., Kong L., Huang W. (2016). Discovery of Novel Hepatitis C Virus NS5B Polymerase Inhibitors by Combining Random Forest, Multiple e-Pharmacophore Modeling and Docking. PLoS ONE.

[25] Hajian-Tilaki K. (2013). Receiver Operating Characteristic (ROC) Curve Analysis for Medical Diagnostic Test Evaluation. Casp. J. Intern. Med..

[26] Poongavanam V., Kongsted J. (2013). Virtual Screening Models for Prediction of HIV-1 RT Associated RNase H Inhibition. PLoS ONE.

[27] Cao S. (2021). Research Onthe Design and Function of Peptide Targeting Aβ1-42 Protein. Master’s Thesis.

[28] Hao J. (2020). Rarional Design, Identification and Application of Affinity Peptide Ligands of Porcine Circovirus Type 2 Cap Protein. PhD’s Thesis.

[29] Hu M. (2020). Antigen-Display Nanoparticles Mediated by Affinity Peptides Targeting Classical Swine Fever Virus E2 Protein and Porcine Circovirus 2 Capsid Protein. PhD’s Thesis.

[30] Wang F., Li N., Wang C., Xing G., Cao S., Xu Q., Zhang Y., Hu M., Zhang G. (2020). DPL: A comprehensive database on sequences, structures, sources and functions of peptide ligands. Database.

[31] Hu M., Wang F., Li N., Xing G., Sun X., Zhang Y., Cao S., Cui N., Zhang G. (2021). An antigen display system of GEM nanoparticles based on affinity peptide ligands. Int. J. Biol. Macromol..

[32] Duffy F.J., Verniere M., Devocelle M., Bernard E., Shields D.C., Chubb A.J. (2011). CycloPs: Generating virtual libraries of cyclized and constrained peptides including nonnatural amino acids. J. Chem. Inf. Model..

[33] O’Boyle N.M., Banck M., James C.A., Morley C., Vandermeersch T., Hutchison G.R. (2011). Open babel: An open chemical toolbox. J. Cheminform..

[34] Kuhn M. (2008). Building Predictive Models in R Using the caret Package. J. Stat. Softw..

[35] Svetnik V., Liaw A., Tong C., Christopher C.J., Sheridan R.P., Feuston B.P. (2003). Random Forest: A Classification and Regression Tool for Compound Classification and QSAR Modeling. J. Chem. Inf. Comput. Sci..

[36] Eric A. (2021). EricArcher/rfPermute, Version 2.5 (v2.5).

[37] Robin X., Turck N., Hainard A., Tiberti N., Lisacek F., Sanchez J.-C., Müller M. (2011). pROC: An open-source package for R and S+ to analyze and compare ROC curves. BMC Bioinform..

[38] Wickham H. (2016). ggplot2: Elegant Graphics for Data Analysis.

